# Matrix stiffness and architecture drive fibro-adipogenic progenitors’ activation into myofibroblasts

**DOI:** 10.1038/s41598-022-17852-2

**Published:** 2022-08-09

**Authors:** Taryn Loomis, Lin-Ya Hu, Ross P. Wohlgemuth, Rosemary R. Chellakudam, Pooja D. Muralidharan, Lucas R. Smith

**Affiliations:** University of California, Davis, CA USA

**Keywords:** Stem cells, Adult stem cells, Stem-cell differentiation, Stem-cell niche, Cell biology, Cell division

## Abstract

Fibro-adipogenic progenitors (FAPs) are essential in supporting regeneration in skeletal muscle, but in muscle pathologies FAPs the are main source of excess extracellular matrix (ECM) resulting in fibrosis. Fibrotic ECM has altered mechanical and architectural properties, but the feedback onto FAPs of stiffness or ECM properties is largely unknown. In this study, FAPs’ sensitivity to their ECM substrate was assessed using collagen coated polyacrylamide to control substrate stiffness and collagen hydrogels to engineer concentration, crosslinking, fibril size, and alignment. FAPs on substrates of fibrotic stiffnesses had increased myofibroblast activation, depicted by αSMA expression, compared to substrates mimicking healthy muscle, which correlated strongly YAP nuclear localization. Surprisingly, fibrosis associated collagen crosslinking and larger fibril size inhibited myofibroblast activation, which was independent of YAP localization. Additionally, collagen crosslinking and larger fibril diameters were associated with decreased remodeling of the collagenous substrate as measured by second harmonic generation imaging. Inhibition of YAP activity through verteporfin reduced myofibroblast activation on stiff substrates but not substrates with altered architecture. This study is the first to demonstrate that fibrotic muscle stiffness can elicit FAP activation to myofibroblasts through YAP signaling. However, fibrotic collagen architecture actually inhibits myofibroblast activation through a YAP independent mechanism. These data expand knowledge of FAPs sensitivity to ECM and illuminate targets to block FAP’s from driving progression of muscle fibrosis.

## Introduction

Fibro-adipogenic progenitors (FAPs) are a mesenchymal-like cell population within skeletal muscle that have the capacity to differentiate down either an adipogenic or myofibroblast lineage. FAPs play a vital role in supporting homeostasis and proper regeneration in skeletal muscle. Under homeostatic conditions, depletion of FAPs results in decreases in the muscle stem cell (MuSC) pool, myofiber size, and functional capacity of muscle^1^. FAPs support tissue repair through expression of key myogenic and immune signals, clearance of necrotic debris, and deposition of extracellular matrix (ECM)^2–4^. Depletion of FAPs causes a regenerative deficiency and induces muscle atrophy, impairing proper muscle function^1,5^. The role of FAPs in acute regeneration is transient with FAPs rapidly proliferating within the first few days post injury, activating into myofibroblasts, depositing ECM, and undergoing macrophage-mediated apoptosis once regeneration is complete^6^. However, in chronic injury, FAPs become resistant to apoptosis and continuously produce ECM components resulting in a more fibrotic tissue^6,7^. Myofibroblast progenitors in other tissues have demonstrated enhanced activation in more stiff fibrotic environments^8^. Thus, FAPs' production of ECM can create a positive feedback loop for progressive fibrosis with increasingly fibrotic tissue leading to an increase in the number of fibrotic FAPs resulting in the deposition of more ECM.

Pathologically, fibrosis is the excess accumulation of ECM components, predominantly collagen. In skeletal muscle, the excess ECM takes the place of contractile material, weakening the muscle and increasing muscle stiffness^9^. Fibrotic material impairs the functional capacity of the muscle, decreasing mobility and leading to contractures^10^. Fibrosis is prevalent across many musculoskeletal diseases such as Duchenne muscular dystrophy, and in cases of severe injury that become chronic^11^. A fibrotic ECM has altered mechanical and architectural properties compared to a healthy ECM, including increased stiffness and crosslinking^12–14^. MuSCs have been shown to be sensitive to their mechanical and architectural environment^15,16^, affecting their capacity for self-renewal and differentiation. Damaged myofibers increase in stiffness causing an increase in MuSC proliferation^17^. Changes in collagen crosslinking and fibril size affect MuSCs’ capacity to differentiate into myotubes^18^. Mesenchymal stem cells (MSCs) are also known to be sensitive to their mechanical and architectural environment^19,20^, although FAPs specifically have not been investigated. Therefore, investigation into FAPs’ response to changes in mechanics and ECM architecture would provide novel insights into these cells’ mechanosensitivity.

FAPs reside in the interstitial space and as a result are in direct contact with the ECM. Collagen, specifically type I collagen, makes up a large portion of the ECM environment, and is the primary load bearing molecule of the ECM^21^. Type I collagen is used in cell culture to treat tissue culture plastic or construct hydrogels. Collagen hydrogels can be manipulated to mimic different aspects of the ECM environment including collagen concentration, crosslinking, fibril size, and fibril alignment^20,22,23^. In this study, we fabricated collagen hydrogels to mimic architectural features of a fibrotic ECM to investigate FAPs’ sensitivity to their architectural and mechanical environment. We hypothesized that there would be an increase in proliferation, myofibroblast activation, and yes-associated protein (YAP) nuclear localization, a measure of mechanosensitivity, on collagen gels that resemble a fibrotic ECM. The goal of this study was to determine if FAPs are sensitive to their mechanical environment and ECM architecture, and if FAPs’ mechanosensitivity plays a role in the development of a regenerative versus fibrotic phenotype.

## Results

### FAP proliferation, myofibroblast activation, and nuclear YAP increase with increasing substrate stiffness

**Figure 1:**
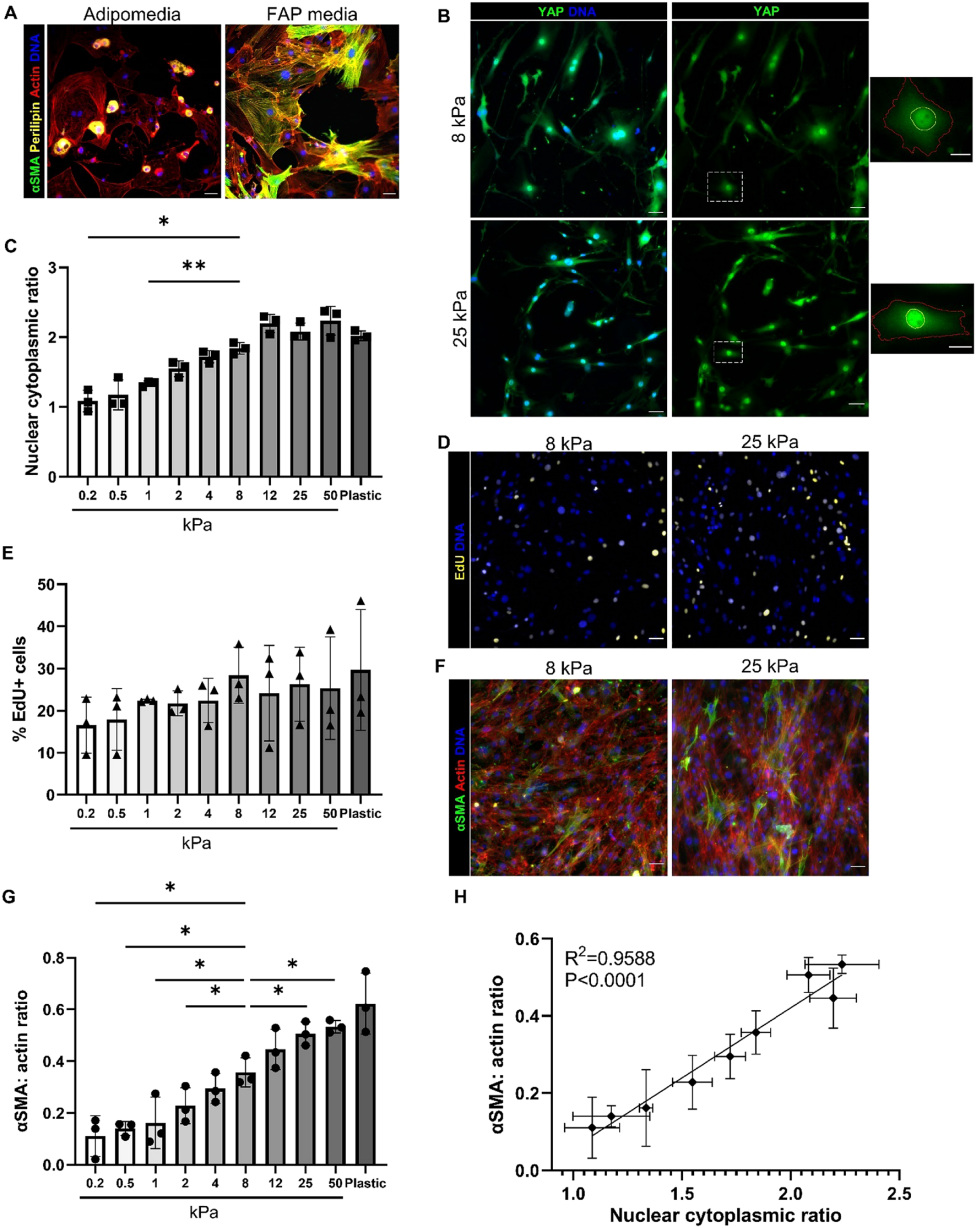
FAPs’ response to substrate stiffness. (**A**) Expression of perilipin and αSMA in FAPs after 7 days in adipogenic or FAP media. (**B**) YAP immunofluorescence of FAPs on polyacrylamide gels on 8 kPa and 25 kPa. Yellow outlines indicate nuclei and red outlines indicate cytoplasm on insets. (**C**) Quantification of YAP signal intensity in nucleus and cytoplasm. (**D, E**) EdU signal and quantification of percent proliferating cells after 24 h EdU treatment (**F**) αSMA signaling as a measure of myofibroblast activation in FAPs. (**G**) Quantification of αSMA and actin area ratio. (**H**) Pearson correlation of αSMA expression and YAP nuclear localization fitted with linear regression line. *P < 0.05, **P < 0.01, compared to 8 kPa via one-way ANOVA using repeated measures with Dunnett correction. Scales bars set to 50 μm. Scale bars for insets set to 20 μm. N = 3 mice and n = 3 independent gels.

FAPs can differentiate into adipocytes or activate into myofibroblasts in vivo. To confirm that these cells maintain their multilineage capacity after primary cell isolation, cells were grown in either adipogenic media or FAP media for 7 days on standard tissue culture plastic. Cells in adipogenic media increased perilipin expression, a marker of lipid droplets and adipogenic differentiation, while cells in the FAP media had increased alpha smooth muscle actin (αSMA) expression (Fig. 1A). Fibrosis is associated with an increase in muscle stiffness. Healthy myofibers are typically 8 kPa compared to fibrotic myofibers that are greater than 18 kPa^16,24^. Therefore, to assess FAPs’ response to changes in substrate stiffnesses that are physiologically relevant, a Matrigen Softwell plate with polyacrylamide hydrogels ranging from 0.2 to 50 kPa was utilized. Polyacrylamide hydrogels were coated with 0.1% collagen to promote cell adhesion. YAP nuclear localization increased with increasing substrate stiffness after 3 days in culture (Fig. 1B,C). Higher substrate stiffness and increased nuclear YAP correlated strongly with each other (Fig. S1A). Therefore, we investigated if early YAP nuclear localization induced changes in FAP behavior, specifically proliferation at 3 days and myofibroblast activation at 7 days. Proliferation rate, measured by EdU incorporation correlated with substrate stiffness, but was not significantly different between any of the gels, although trended upward with increasing stiffness (Fig. 1D,E, Fig. S1B). Myofibroblast activation was significantly higher on substrates with fibrotic stiffnesses compared to a physiologically, healthy modulus (Fig. 1F,G). The increase in myofibroblast activation was strongly correlated with the increase in substrate moduli, indicating FAPs ability to detect their underlying substrate mechanics (Fig. S1C). The increase in myofibroblast activation and YAP nuclear localization were highly correlated indicating nuclear YAP may be driving this change cell phenotype(Fig. 1H). Recent studies have shown a subpopulation of FAPs marked by high Vcam1 expression is significantly increased during injury and fibrosis in mice^25^. To investigate if the increase in myofibroblast activation is driven by Vcam1 positive FAPs, we looked at Vcam1 and αSMA expression in FAPs cultured on substrates of increasing stiffness. There were similar levels of Vcam1 expression in αSMA+ and αSMA− FAPs (Fig. S2A,B). As opposed to myofibroblast activation, the adipogenic differentiation of FAPs over 7 days was not sensitive to the range of substrate stiffnesses tested (Fig. S2C). Overall, FAPs appear sensitive to changes in substrate stiffness through YAP localization and associated myofibroblast activation, although substrate stiffness did not impact Vcam1 expression nor adipogenesis.

### YAP localization sensitive to collagen concentration

**Figure 2:**
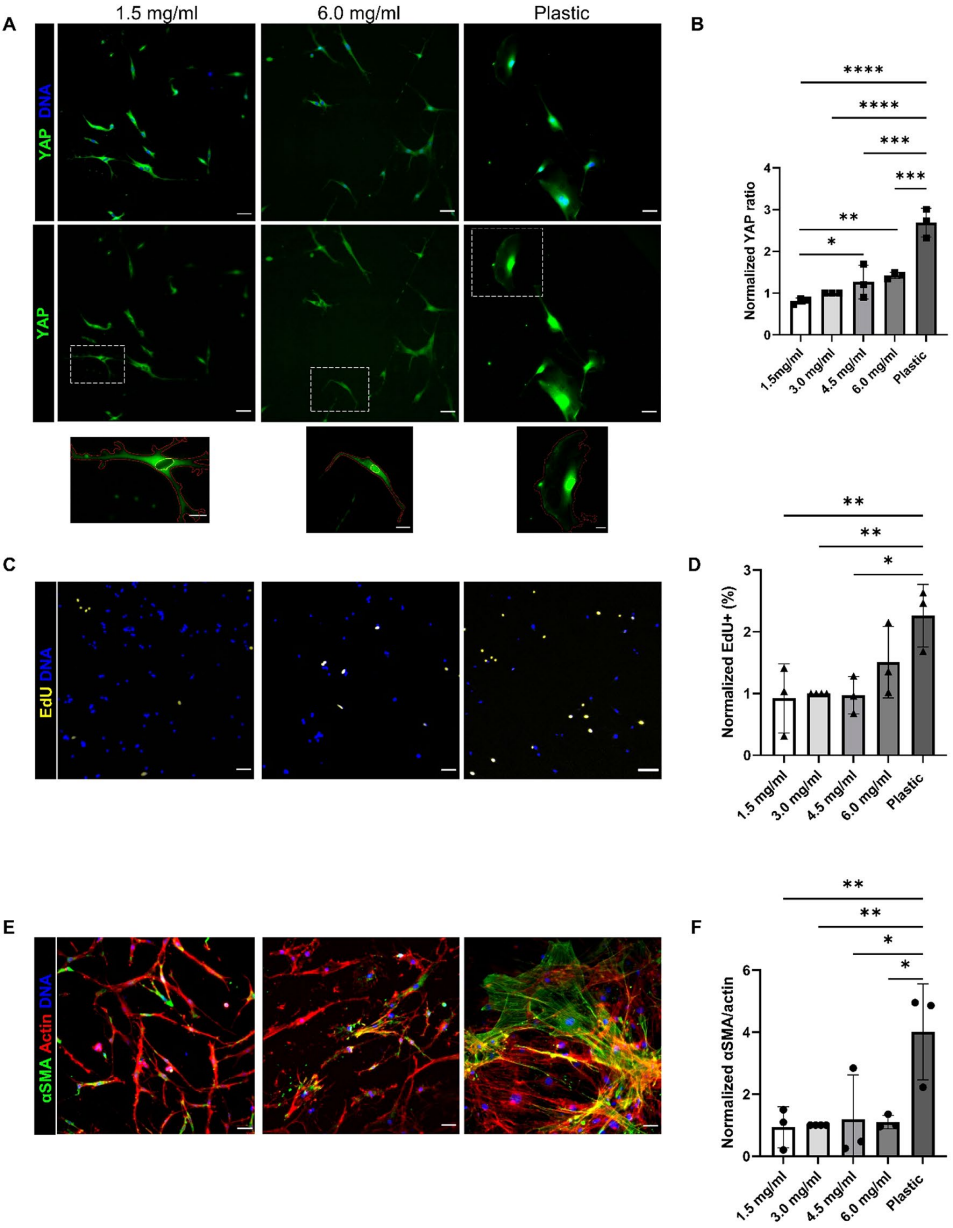
FAP behavior in response to scaling collagen concentration. (**A**) YAP immunofluorescence of FAPs on 1.5 and 6.0 mg/ml collagen gels and tissue cultured plastic. Yellow outlines indicate nuclei and red outlines indicate cytoplasm on insets. (**B**) Quantification of YAP signal intensity as a nuclear to cytoplasmic ratio. (**C, D**) EdU signal and quantification across collagen concentrations and plastic. (**E**) αSMA signaling in FAPs after 7 days in FAP media. (**F**) Quantification of αSMA and actin area ratio as a measure of myofibroblast activation. *P < 0.05, **P < 0.01, ***P ≤ 0.001, ****P < 0.0001, One-way ANOVA with multiple comparisons with Dunnett correction. Data normalized to 3.0 mg/ml. Scales bars set to 50 μm. Scale bars for insets set to 20 μm. N = 3 and n = 3 for 1.5, 4.5, 6.0 mg/ml and plastic. N = 4 and n = 4 for 3.0 mg/ml. N = number of mice, n = number of independent gels.

Fibrosis not only alters the mechanics of the ECM, but also the ECM content and architecture^11,12^. Since fibrosis is associated with an increase in ECM components, specifically collagen, we investigated FAPs’ response to telocollagen gels with increasing collagen concentrations of 1.5, 3.0, 4.5 and 6.0 mg/ml. FAPs were also grown on tissue cultured plastic, which represents a rigid substrate compared to the soft collagen gels. YAP nuclear localization was sensitive to changes in collagen concentration and stiffness (Fig. 2A,B). Proliferation was not affected by collagen concentration but increased on plastic compared to all the collagen gels except the 6.0 mg/ml gels (Fig. 2C,D). There was no difference in myofibroblast activation across the range of collagen gels, but activation did increase on plastic compared to all the gels (Fig. 2E,F). Cellular and nuclear area were not significantly different across the substrate conditions, although cells on plastic tended to be more spread out and have higher cell density (Fig. S3A–C). Overall, YAP nuclear localization was affected by collagen concentration, but not to an extent that altered FAP proliferation or activation into myofibroblasts.

### Collagen crosslinking inhibits FAP activation into myofibroblasts

**Figure 3:**
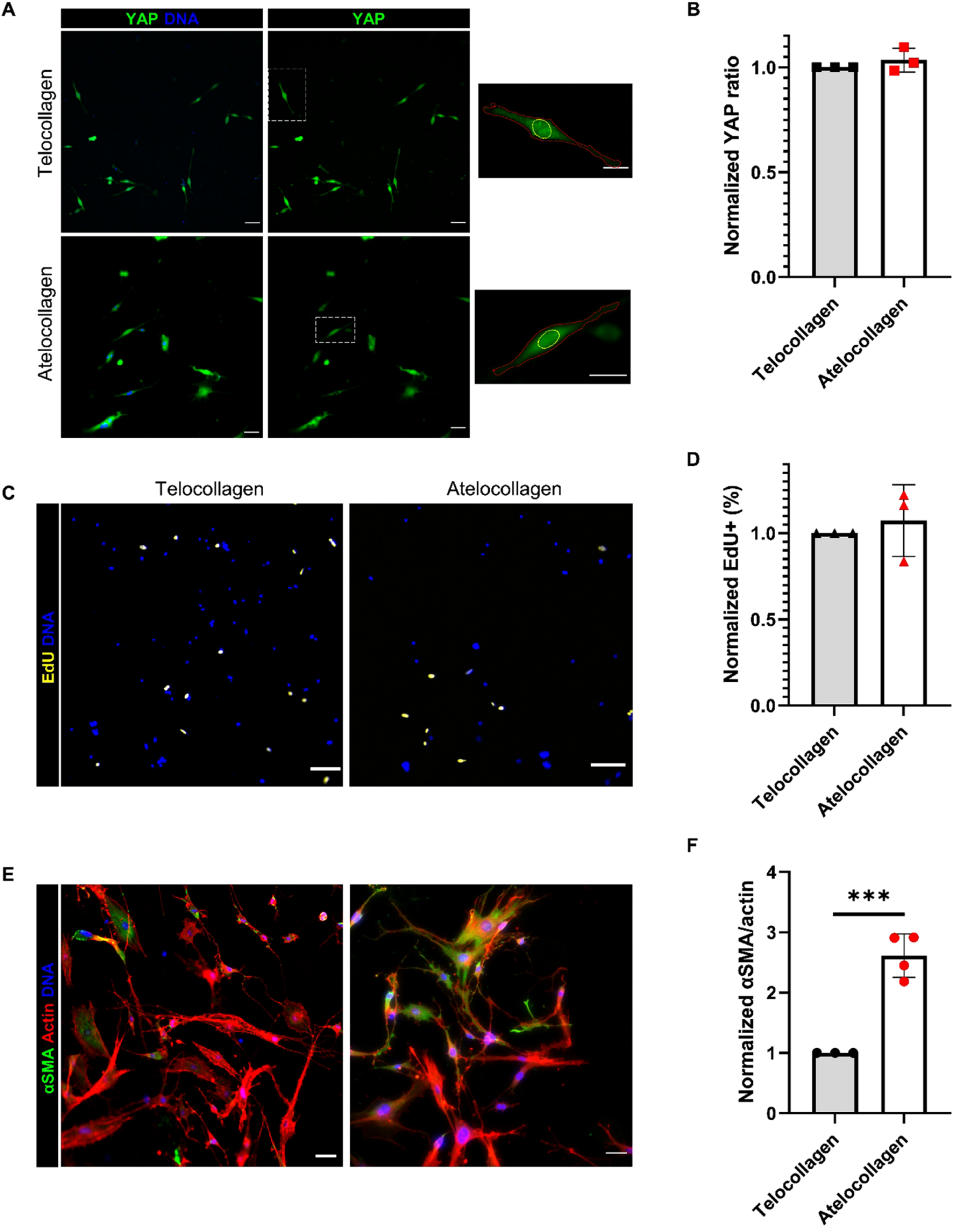
FAPs’ response to 3 mg/ml crosslinked and non-crosslinked collagen gels. (**A**) YAP immunofluorescence of FAPs on telocollagen and atelocollagen gels. Yellow outlines indicate nuclei and red outlines indicate cytoplasm on insets. (**B**) Quantification of YAP signal intensity to find nuclear to cytoplasmic ratio of YAP. (**C, D**) EdU signal and quantification of percent proliferating cells after 24 h EdU treatment (**E**) αSMA signaling as a measure of myofibroblast activation in FAPs. (**F**) Quantification of αSMA and actin area ratio. ***P < 0.001. Data normalized to telecollagen gels. Scales bars set to 50 µm. Scale bars for insets set to 20 µm. N = 3 mice and n = 3 independent gels.

Further investigation was conducted into several aspects of collagen architecture. Collagen crosslinking is known to increase in fibrotic conditions, including muscle fibrosis^14^. Pyridinoline crosslinking of collagen fibrils occurs in the telopeptide region of the fibril^26,27^. Atelocollagen is a collagen solution with the telopeptide region cleaved, preventing crosslinking. Atelocollagen was used alongside telocollagen to evaluate the effect of collagen crosslinking on FAP behavior. YAP localization was not affected by crosslinking (Fig. 3A,B). Proliferation rate did not change between telocollagen and atelocollagen (Fig. 3C,D). Myofibroblast activation significantly increased when crosslinking was prevented (Fig. 3E,F). Surprisingly, this data demonstrates that collagen crosslinking blocks FAPs activation into myofibroblasts without a significant impact on proliferation or YAP localization.

### FAPs deform the collagen matrix

**Figure 4:**
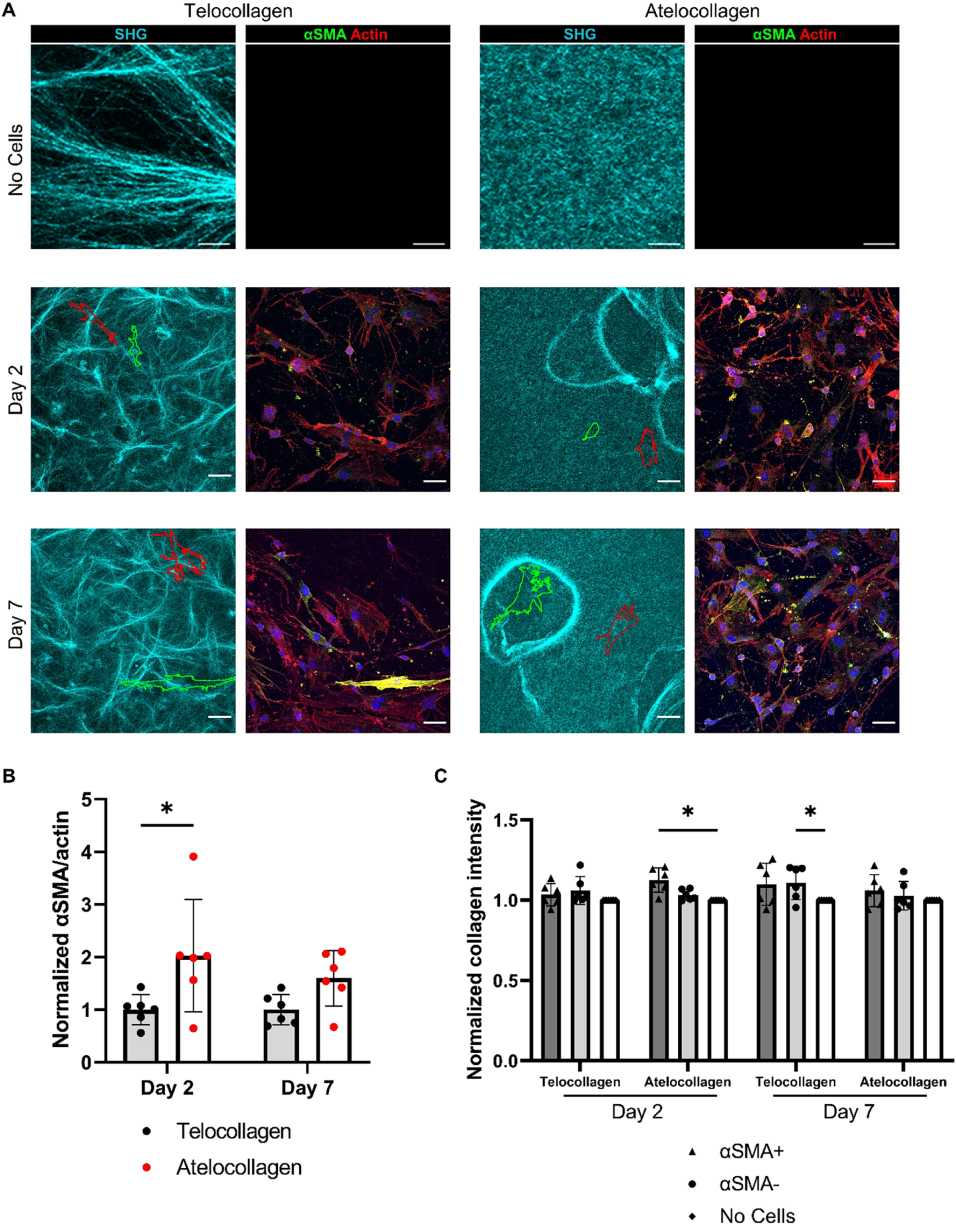
SHG imaging of 3 mg/ml crosslinked and non-crosslinked collagen gels. (**A**) SHG images of collagen and FAP cells. Red outlines indicate a αSMA− FAP and green outlines indicate a αSMA+ FAP. Scale bars on no cell images set to 10 μm. Scales bars on day 2 and day 7 images set to 50 µm. (**B**) αSMA and actin area ratio after 2 and 7 days on the collagen gels. (**C**) Collagen intensity of gels under αSMA+ or αSMA− cells, or under no cells normalized to areas of collagen without any cells. *P < 0.05 using a two-way ANOVA with multiple comparisons using Tukey correction. N = 2 independent gels and n = 3 fields per gel.

The decrease in myofibroblast activation with collagen crosslinking was unexpected due to the increase in crosslinking seen in fibrosis^12,14^. We hypothesized that this disparity was due to the increased ability of the FAPs to deform the atelocollagen, creating localized areas of higher stiffness and collagen content. To investigate this, we imaged telocollagen and atelocollagen gels using second harmonic generation (SHG) imaging after 2 and 7 days with FAPs to observe changes in collagen distribution across the gel. We hypothesized due to the contractility of myofibroblasts, these cells would be able to deform the matrix more than non-activated FAPs. Therefore, we looked at αSMA+ and αSMA− FAPs independently to observe differences in collagen distribution. There was an overall increase in collagen intensity under the cells, significantly on the day 2 atelocollagen and day 7 telocollagen gels. However, there was no difference in collagen intensity between at αSMA+ and αSMA− FAPs (Fig. 4A,C).

These cells showed consistency with previous myofibroblast activation results (Fig. 4B). However, there was no significant difference in collagen intensity between the atelocollagen and telocollagen gels, suggesting the cells were not deforming the atelocollagen gels to a greater degree.

### Larger collagen fibrils inhibit FAP activation into myofibroblasts

**Figure 5:**
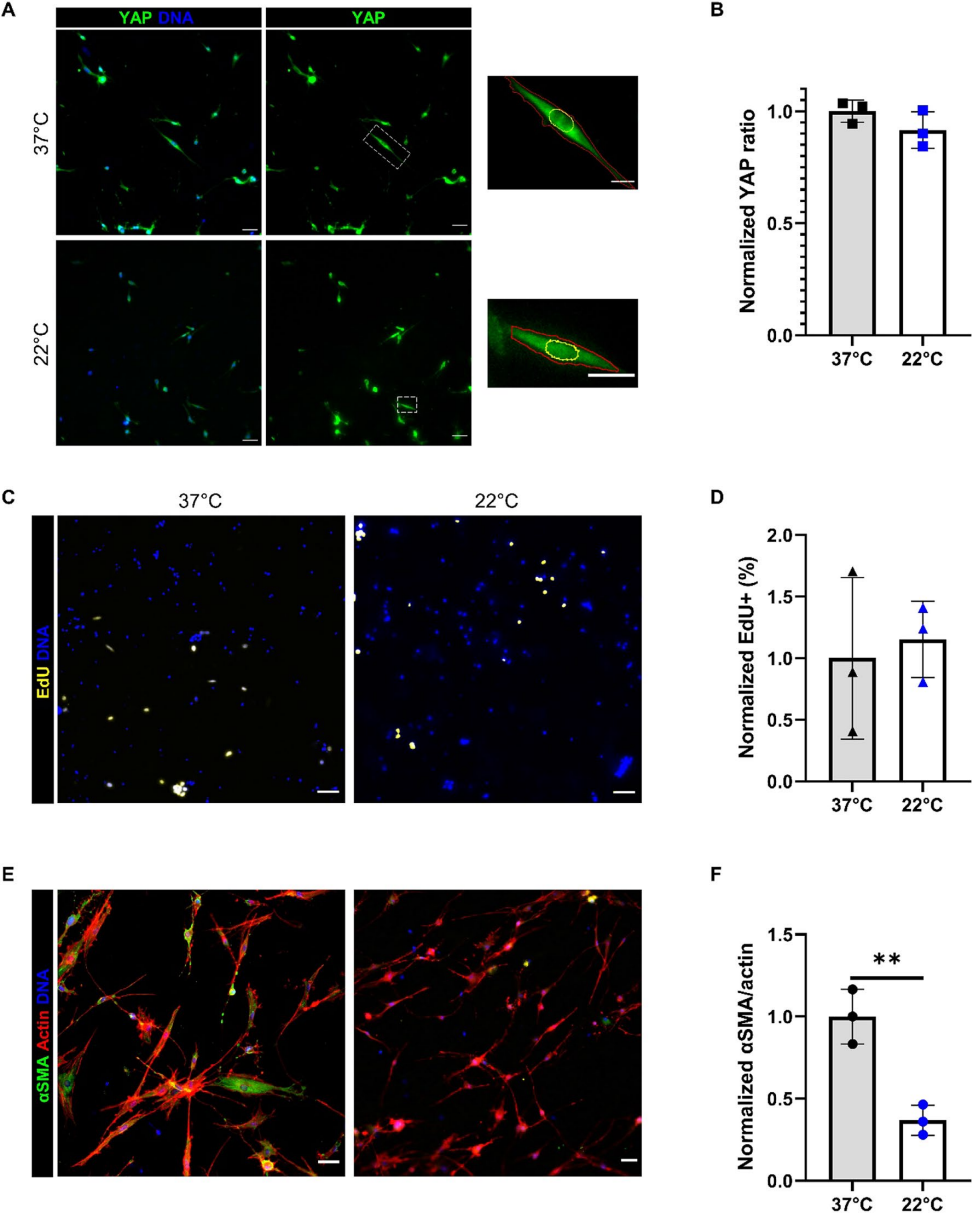
Effect of fibril size on FAP behavior. (**A**) YAP immunofluorescence of FAPs on 3 mg/ml telocollagen gels polymerized at 37 and 22 °C. Yellow outlines indicate nuclei and red outlines indicate cytoplasm on insets. (**B**) Quantification of YAP signal intensity to find nuclear to cytoplasmic ratio of YAP. (**C, D**) EdU signal and quantification of percent proliferating cells after 24 h EdU treatment. (**E**) αSMA signaling in FAPs after 7 days. Scales bars set to 50 μm. (**F**) Quantification of αSMA and actin area ratio. **P < 0.01. Data normalized to 37 °C. Scales bars set to 50 µm; insets set to 20 µm. N = 1 mouse and n = 3 independent gels.

Along with the abundance of collagen fibers in fibrosis is a shift to collagen fibers and fibrils of a larger diameter^28^. Collagen fibril size in hydrogels is affected by polymerization temperature with a higher temperature resulting in more fibrils of smaller sizes than lower temperatures^29,30^. Collagen gels were polymerized at 22 °C along with 37 °C to elucidate FAPs response to fibril size. Proliferation and YAP localization were not changed between the two conditions (Fig. 5A–D). Smaller collagen fibrils resulted in significantly increased myofibroblast activation compared to the larger fibrils (Fig. 5E,F). As with collagen crosslinking, surprisingly, increased collagen fibril size appeared to inhibit FAP activation into myofibroblast.

### FAPs deform smaller collagen fibrils

**Figure 6:**
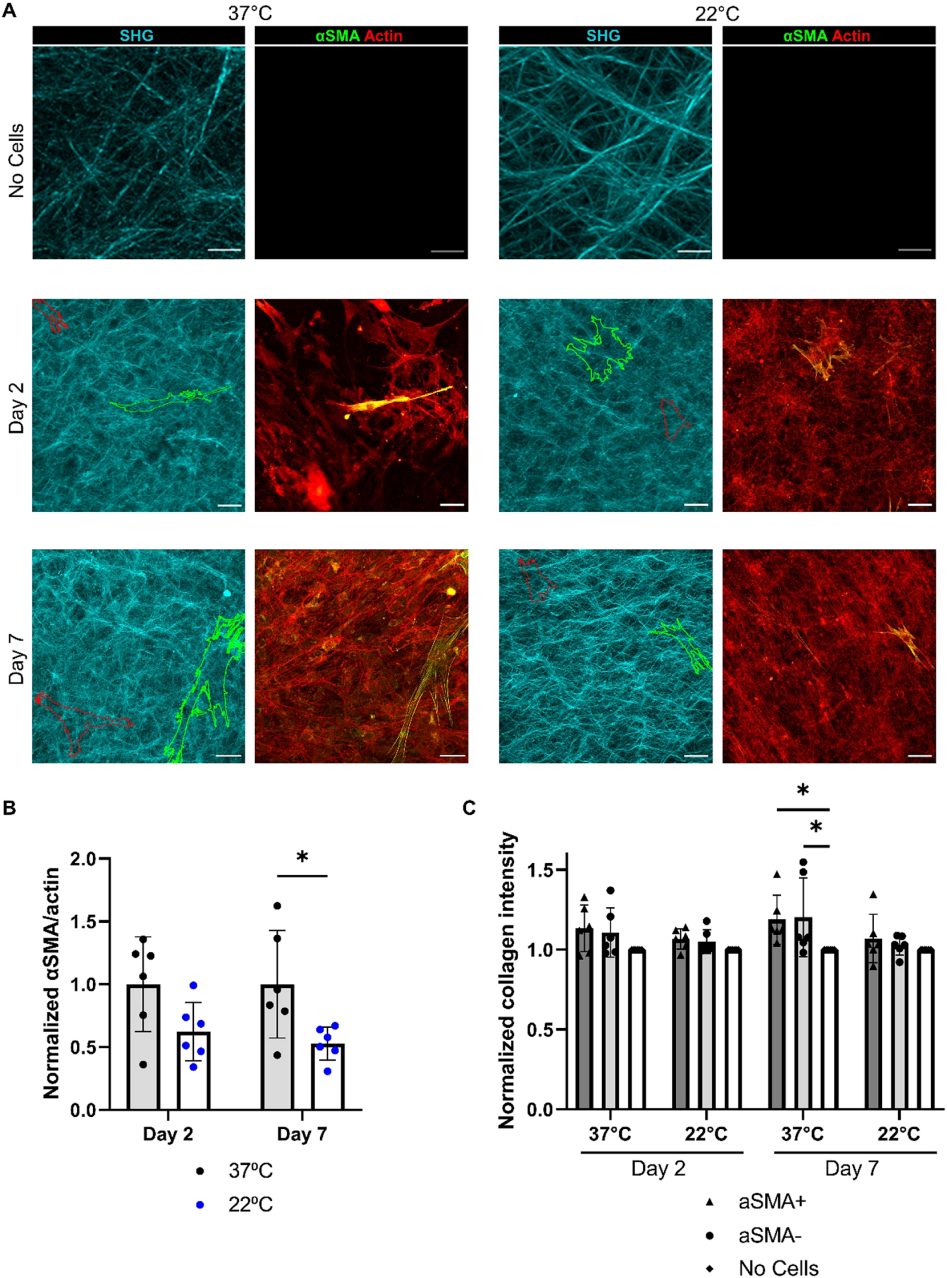
SHG imaging of 3 mg/ml collagen gels polymerized at different temperatures. (**A**) SHG images of collagen gels polymerized at 37 °C and 22 °C at day 2 and day 7 after cell seeding. Scale bars on no cell images set to 10 μm. Scales bars on day 2 and day 7 images set to 50 μm. Red outlines indicate a αSMA− FAP and green outlines indicate a αSMA+ FAP. (**B**) Myofibroblast activation at day 2 and day 7 across the gels. (**C**) Collagen intensity under no cells, αSMA+, or αSMA− FAPs. Intensity normalized to areas of gels without cells. *P < 0.05 using a two-way ANOVA with multiple comparisons using Tukey correction. N = 2 independent gels and n = 3 fields per gel.

Smaller collagen fibrils are more deformable and malleable to cell deformation^20^. We investigated if the FAPs were deforming the collagen gels polymerized at 37 °C more than those polymerized at 22 °C, which may have contributed to the differences in myofibroblast activation. SHG imaging of the collagen gels revealed an increase in collagen intensity under cells on the 37 °C gels compared to areas without cells. This suggests that the cells were able to pull the matrix and localize the collagen. There was no significant difference in collagen distribution across the 22 °C gels (Fig. 6A,C). The decrease in myofibroblast activation on the 22 °C gels was consistent with previous results (Fig. 6B). It appeared that the FAPs, regardless of whether activated into myofibroblasts or not, were able to deform the 37 °C collagen gels but not the 22 °C gels. Together, with the data on myofibroblast activation on non-crosslinked gels, this implicates substrate malleability as a potential factor in myofibroblast activation.

### Collagen alignment does not impact FAP proliferation, differentiation, or YAP localization

Collagen gels were aligned using magnetic beads during initial stages of polymerization^22^. FAPs were plated on aligned or unaligned telocollagen gels to determine the effect of alignment on FAP behavior. FAPs were able to sense their underlying substrate and elongate their nuclei along the axis of collagen alignment (Fig. S4A,B). Proliferation and YAP nuclear localization was consistent across the two conditions after 2 days (Fig. S4C–F). Myofibroblast activation did not change with alignment over the course of a week (Fig. S4G,H). Skeletal muscle is typically highly anisotropic^12,31^, and FAPs showed preference to align their nuclei with the direction of the fibrils, but that alignment did not significantly impact the FAP response in the other measures investigated.

### YAP inhibition reduces myofibroblast activation on stiff substrates

**Figure 7:**
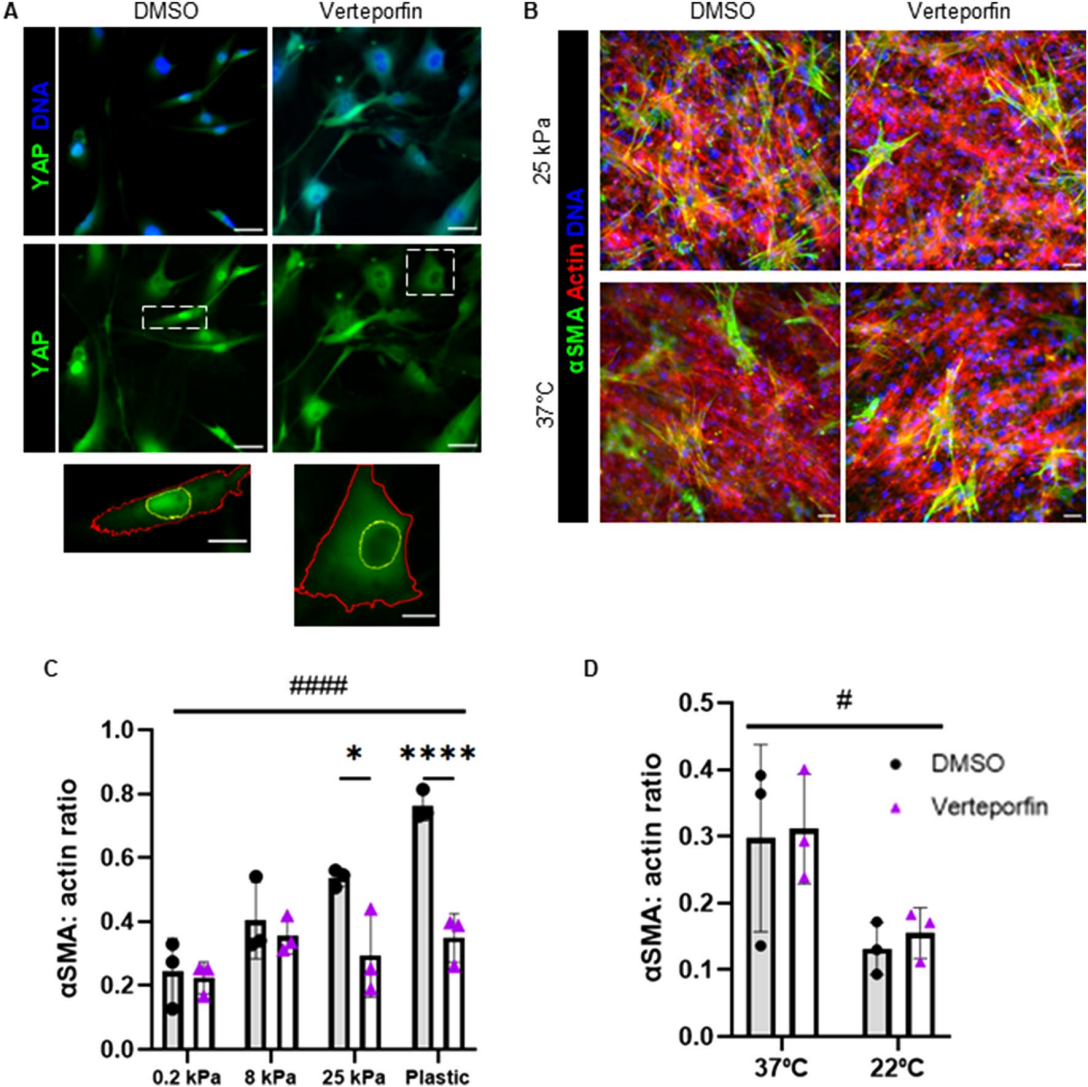
Yap inhibition reduces myofibroblast activation. (**A**) YAP localization in FAPs cultured on plastic in DMSO or 0.5 µM verteporfin for 2 days. Scale bars are 50 µm, insets are 20 µm. Yellow outlines indicate nuclei and red outlines indicate cytoplasm on insets. (**B**) Myofibroblast activation in FAPs cultured for 7 days with 0.5 µM verteporfin or 0.1% DMSO on collagen-coated polyacrylamide gels at 25 kPa and 3.0 mg/ml telocollagen gels polymerized at 37 °C. Scale bars set to 50 µm. (**C**) Quantification of myofibroblast activation of FAPs in DMSO or 0.5 µM verteporfin on collagen-coated polyacrylamide gels. (**D**) Quantification of myofibroblast activation of FAPs in DMSO or 0.5 µM verteporfin on collagen gels polymerized at different temperatures. *P < 0.05, ****P < 0.0001 between DMSO and verteporfin treatment. #*P* < 0.05, ####*P* < 0.0001 between substrates.

Given the strong correlation between YAP nuclear localization and myofibroblast activation on stiff substrates, we investigated if blocking YAP activity would reduce αSMA expression in FAPs. FAPs were cultured on collagen-coated polyacrylamide gels at 0.2, 8, 25 kPa, and plastic as well as on collagen gels polymerized at 37 °C and 22 °C. There was a significant increase in αSMA expression with increasing substrate stiffness. With the addition of verteporfin, an inhibitor of YAP activity, YAP localization to the nucleus decreased (Fig. 7A). After 7 days, verteporfin led to the elimination of mechanosensitivity as αSMA expression was significantly reduced in FAPs on the 25 kPa gel and on plastic (Fig. 7B,C). However, blocking YAP did not impact FAP sensitivity to collagen fibril diameter as verteporfin did not appear to have an effect on FAPs cultured on collagen gels polymerized at different temperatures (Fig. 7B,D).

## Discussion

The goal of this study was to investigate FAPs’ sensitivity to their underlying substrate mechanics and architecture. The mechanosensitivity of FAPs has not, to our knowledge, been investigated. Sensitivity to their mechanical and architectural environment may play a key role in FAP’s contribution to fibrosis, with fibrotic muscle inducing a more fibrotic FAP phenotype. We focused on proliferation and myofibroblast activation as measures of a fibrotic phenotype and YAP localization as a potential mechanosensing pathway. Polyacrylamide hydrogels with collagen coating were used to investigate a range of physiologically relevant stiffnesses. Collagen gels were engineered to manipulate collagen concentration, crosslinking, fibril size, and alignment. YAP nuclear localization increased on substrates with increasing stiffness. Proliferation was increased on plastic compared to collagen gels but was not affected by architectural changes in substrate. Myofibroblast activation was inhibited on gels with crosslinking, larger fibrils, or lower stiffness. Alignment affected the orientation of nuclei but had no significant effect on proliferation or myofibroblast activation. Increased YAP nuclear localization correlated with higher myofibroblast activation levels when associated with altered mechanics. However, YAP localization was not correlated with changes induced via architecture. Using verteporfin to block YAP activity, resulted in a reduction of myofibroblasts on stiff substrates but not on substrates with altered architecture. Overall, this study demonstrates that FAPs are sensitive to aspects of both their mechanical and architectural environment with implications for their role in propagating fibrosis.

Fibrosis is associated with an increase in tissue stiffness, with skeletal muscle bundles increasing from around 8 kPa in healthy muscle to > 20 kPa in fibrosis^11,16^. To investigate if this change in stiffness altered FAP behavior, collagen-coated polyacrylamide gels ranging from 0.2 to 50 kPa were utilized. YAP nuclear localization and myofibroblast activation scaled with increasing stiffness. There were significant differences in myofibroblast activation between 8 and 25 kPa. The increase in YAP nuclear localization was highly correlated with the increase in myofibroblast activation indicating that YAP may be driving this change. Previous studies have shown that YAP nuclear localization is tied to fibroblast activation and proliferation^32–34^. Blockage of YAP activity with verteporfin reduced myofibroblast activation on 25 kPa gels and on plastic. This supports previous studies that have shown a reduction in YAP activity, αSMA expression, and fibrosis with the addition of verteporfin^32,35,36^. The increase in myofibroblast activation supports the concept of a feedforward pathway of fibrosis. A fibrotic environment induces FAP activation into myofibroblasts, which in turn increases ECM deposition making the tissue increasingly fibrotic^3,33^. The expression of Vcam1 is significantly upregulated in FAPs during injury and fibrosis, suggesting it may play a role in fibrotic development^25^. However, Vcam1 levels were similar across FAPs cultured on different stiffness and between αSMA+ and αSMA− cells, suggesting Vcam1 is not driving the changes induced by stiffness. Due to the limited availability of primary FAPs, cells were cultured on tissue-cultured plastic for 1–2 weeks before plating on substrates of altered stiffness. It is possible that this time on rigid plastic induced activation of Vcam1 across all cells that was maintained even on soft substrates. In addition to driving production of fibrotic material as myofibroblasts, FAPs in diseased conditions also undergo adipogenesis leading to fatty infiltration of muscle. While we hypothesized this would be supported on softer substrates in adipogenic media there was no difference in the FAP produced adipocytes with stiffness. However, mechanosensitivity of adipogenesis is largely regulated by cyclic strain which was not incorporated into our system^37^. This is the first study to specifically demonstrate FAP’s substrate mechanosensitivity, with stiff environments reinforcing YAP associated pathways toward myofibroblasts.

The ECM architecture, as well as mechanics, are altered in a fibrotic muscle. Fibrosis is largely tied to an increase in ECM content, specifically collagen. Therefore, we manipulated collagen hydrogels to mimic different aspects of the ECM architecture altered in fibrosis that FAPs residing in the interstitial matrix would experience. The range of collagen concentrations investigated in this study are representative of in vivo concentrations, although the in vivo organization of collagen fibrils into cables is not reconstituted in vitro^26,38,39^. While the modulus of the collagen gels scaled with concentration, overall, the stiffnesses of the gels (0.1–0.4 kPa) were well below the physiological range^11,18^. YAP nuclear localization scaled with increasing collagen concentration and therefore, stiffness. However, this change in YAP did not correlate well with changes in proliferation or myofibroblast activation. As YAP translocates to the nucleus in response to increased substrate stiffness, there may be a concentration threshold needed to observe phenotypic changes. This threshold is reached on plastic, where the significant increase in nuclear YAP compared to collagen gels correlated with an increase in proliferation and myofibroblast activation.

Collagen crosslinking increases in skeletal muscle fibrosis^12,14^. Surprisingly, myofibroblast activation increased with the removal of collagen crosslinking. Collagen stiffness and crosslinking are often associated with each other, but studies have suggested they do not strongly correlate in skeletal muscle^40^. The stiffness of the collagen gels used here has been shown to remain relatively similar with or without crosslinking, which is supported by similar YAP nuclear to cytoplasmic ratios across the gels^18,40^. This suggests that the increase in myofibroblast activation may be driven by a signaling pathway independent of YAP. Crosslinked collagen gels have been shown to have increased resistance to deformation when compared to gels without crosslinking due to the contraction of the crosslinked collagen fibrils^41^. Despite increased resistance to deformation, there was no significant difference in matrix remodeling between the atelocollagen and telocollagen gels. FAPs did appear to pull the matrix around them regardless of cell fate. MSCs have improved differentiation on reduced crosslinked collagen substrates due to an enhanced ability of cells to contract^42^. FAPs may activate into myofibroblasts on the atelocollagen gels to compensate for the lack of crosslinks by producing a more crosslinked matrix. TGF-β is a predominant driver of fibrosis in vivo*,* inducing the activation of FAPs into myofibroblasts and causing the cells to be resistant to apoptosis. Soluble factors, such as TGF-β signaling, may dominate over the architectural cues from crosslinking in fibrotic tissue^6^. Contrary to our initial hypothesis, this result implicates a YAP and stiffness independent mechanism of myofibroblast activation that is diminished in response to collagen crosslinking.

To investigate the effect of fibril size, collagen gels were polymerized at 22 °C, to produce larger fibrils, compared to the standard gel formed at 37 °C^29^. MSCs are known to have increased proliferation and increased adipogenic differentiation on collagen gels polymerized at 37 °C compared to 22 °C^20^. Similarly, our study showed that gels with higher polymerization temperatures resulted in more myofibroblast activation but did not change their proliferation rate. This increase in myofibroblast activation was not correlated with changes in YAP localization, and the inhibition of YAP activity did not appear to have any effect on αSMA expression. This indicates that the increase in myofibroblast activation is driven by a YAP-independent mechanism. The change in myofibroblast activation may be due to an increase in intrafibrillar crosslinking at 22 °C due to the larger fibril diameter. Higher polymerization temperatures result in smaller, longer fibrils allowing for increased focal adhesions and cell spreading^29,30,43^. Despite no changes in overall bulk stiffness, fibrils polymerized at higher temperatures are more flexible and malleable to cell deformation^18,20^. MSCs that can remodel their substrate have increased cell spreading, which is linked to the expression of myofibroblast markers including αSMA^20,44,45^. In this study, we found that FAPs may be able to deform the collagen matrices with smaller fibrils, which correlated to higher αSMA expression. Alternatively, the increase in collagen intensity may also be caused by an increase in collagen secretion from FAPs on the gels polymerized at higher temperatures. FAPs’ activation into myofibroblasts may be driven not only by mechanical and architectural sensing, but also the cells’ ability to remodel the surrounding matrix.

Another key aspect of collagen architecture is alignment of fibrils. Collagen fibrils are highly aligned in skeletal muscle and altered in fibrosis^12^. Alignment of collagen fibrils does not affect overall stiffness of the gels, resulting in similar YAP localization^18,22^. ECM alignment is important in skeletal muscle for force transmission and proper muscle fiber regeneration. Despite the highly aligned nature of skeletal muscle, there was no change in FAP behavior with alignment except for the cells orienting their nuclei along the axis of alignment. MSCs grown on collagen gels showed no difference in osteogenic or adipogenic differentiation with or without collagen alignment despite cells orienting with the direction of the fibrils^46^. While alignment of the ECM is important in regeneration to promote properly aligned myofibers, it does not appear to affect FAPs’ cell fate.

Collagen hydrogels provide an opportunity to investigate in vivo changes in architecture, yet also pose several limitations. FAPs reside in the interstitial space in vivo and are completely surrounded by ECM^47^. This study investigated FAPs’ response to collagen architecture in a 2D environment rather than a 3D environment, which may have influenced cell–matrix interactions^48–50^. This in vitro study allows us to isolate the influence individual aspects of the ECM architecture play on FAPs but does not reconstitute the complex in vivo environment. While we have demonstrated stiffness, collagen crosslinking, and fibril size are capable of dictating FAPs’ response, how these signals are interpreted among the complexity of the environment in vivo requires further study. Additionally, due to limited number of FAPs isolated from a single dissection, cells were grown up on tissue cultured plastic before being passaged to the collagen gels for the myofibroblast activation assay. Previous reports have shown different cell lines, including adipose stem cells and MSCs, retain mechanical memory of their initial substrate stiffness^51,52^. MSCs, which share many similarities to FAPs, have been shown to maintain mechanosensitivity despite mechanical memory^52^. Additionally, the differences observed in myofibroblast activation across the collagen gels, despite being cultured on plastic first, indicate that FAPs do retain mechanosensitivity regardless of potential mechanical memory. The results of this study have implications in musculoskeletal diseases such as muscular dystrophies where fibrosis is prevalent. FAPs play a central role in the development and progression of fibrosis. The development of fibrotic ECM provides mechanical and architectural cues to FAPs to further fibrosis through activation into myofibroblasts. YAP appears to dictate FAPs’ response to changes in mechanics, but not the response to architecture suggesting another signaling pathway drives architectural sensing. While more research is needed to uncover this alternative pathway, it could represent a target for novel anti-fibrotic therapy. The ECM is an important target in treating fibrosis to make the environment more conducive to both a regenerative FAP phenotype and MuSC regeneration.

## Conclusions

Overall, this study provides novel insights into the mechanosensitivity of FAPs, which has not to our knowledge been investigated. This study shows that FAPs’ sensitivity to their substrate expands beyond mechanics to architecture as well. YAP has been a well-known target for mechanosensitivity investigations. Yet, in this study, we show that YAP does not correlate with changes induced by substrate architecture. This study provides novel therapeutic ECM targets to modulate FAP behavior in fibrosis.

## Methods

### Animal handling

Mice were obtained from Jackson Laboratory and bred at a UC Davis animal facility. The animals were given food and water ad libitum and kept on a 12-h light/dark cycle. Wild-type male and female mice aged 6–12 weeks were used for primary cell collection, animals were selected randomly from the pool of cages. CO_2_ followed by cervical dislocation was used to euthanize the animals prior to muscle collection. All animal experiments were in compliance with ARRIVE guidelines, approved by the University of California Davis Institutional Animal Care and Use Committee, and performed in accordance with the relevant guidelines and regulations.

### FAP isolation and culture

To isolate FAPs, the lower limb skeletal muscles of wild type mice were dissected. Muscle tissue pieces were digested in an enzyme mix from Skeletal Muscle Dissociation Kit (Miltenyi Biotec) in two 30 min intervals at 37 °C under continuous rotation. Mechanical disruption via a tissue homogenizer was used between intervals. The digested muscle solution was filtered using a 70 µm filter, and red blood cell lysis solution was added to remove erythrocytes. CD140a (PDGFRα) magnetic beads (Miltenyi Biotec 130-101-502) were added to filtered cells to label FAPs for positive selection through a MACs Column and Separator (Miltenyi Biotec). The isolated FAPs were added to either tissue culture treated plastic plates or directly onto collagen gels in FAP media (DMEM, 20% FBS (Biowest S1620), 10% HS (Fisher SH3007403) , 1% Penicillin–Streptomycin (Fisher 15-140-122), 50 ng/ml bFGF (Fisher PHG6015)). For adipogenic differentiation, cells were grown in DMEM containing 10% FBS, 1% Penicillin–Streptomycin, 0.25 µM dexamethasone (Fisher 1126100), 0.5 mM IBMX (VWR 102516-252), 5 µM troglitazone (Fisher 501150786) , and 1 µg/mL insulin (Fisher MP219390025) for 7 days^2^. Cells were fed with fresh media every 2–3 days.

### Collagen gel fabrication

Glass bottom petri dishes were treated before the addition of collagen gel solution to ensure collagen adherence^53^. Dishes were treated by adding 2% aminopropyltriethoxysilane (Sigma A36648) for 15 min, washing with deionized water for 5 min, air-drying for 15 min, 0.1% glutaraldehyde (Sigma G5882) for 15 min, and rinsing with deionized water three times. Collagen gel solutions were prepped over ice to prevent polymerization before addition to treated dishes. Solutions were made with either telocollagen (Telecol 10, Advanced Biomatrix 5226) or atelocollagen (Fibricol, Advanced Biomatrix 5133) and diluted to desired concentration 10% 10X PBS diluted with deionized water to 1X PBS and 1N NaOH to a pH of 7.0. All collagen gels were made at a concentration of 3.0 mg/ml except in the case of altered collagen concentrations. Aligned collagen gels were prepared with the addition of 1% magnetic Polystyrene magnetic particles (Spherotech PM-20-10)^22^. Collagen solutions were pipetted onto treated glass dishes and topped with a glass coverslip to ensure a flat surface for cell adhesion and imaging. Collagen gels were left to polymerize at either 37 °C for 90 min or 22 °C for 4 h. Aligned collagen gels were placed against a strong magnet for to pull beads in one direction, which took less than 1 min, and then transferred to 37 °C to finish polymerization. After polymerization, coverslips were removed, and gels were either used immediately or stored in 1X PBS at 4 °C for up to 3 weeks. Unaligned, 3 mg/ml telocollagen gels polymerized at 37 °C were considered the control across each experimental condition. A Softwell 96-well plate with polyacrylamide hydrogels ranging in stiffnesses from 0.2 to 50 kPa was utilized (Matrigen). Hydrogels were coated in collagen at a concentration of 0.1% telocollagen, left to polymerized at 37 °C, and then excess solution was removed before use.

### EdU assay

FAPs were seeded on collagen gels in a 6 mm cloning cylinder in FAP media at a concentration of 35,000 cells/mm^2^ immediately following cell isolation. Cells were given 48 h to adhere, and then the media was replaced with fresh FAP media plus 0.1% EdU (Invitrogen). Cells were incubated with EdU for 24 h, and then fixed with 4% paraformaldehyde (Fisher AA433689M). Cells were labeled for EdU incorporation with a solution of 100 mM Tris (Fisher BP152 500), 1 mM CuSO4 (Fisher 60-045-185) , 10 µM Alexa647-Azide (Fisher A10277) , and 100 mM ascorbic acid (Fisher AC401475000) for 30 min.

### Myofibroblast activation assay

FAPs were cultured on tissue-cultured plastic for 7 days before assay to ensure high enough cell count. FAPs were then seeded on collagen gels in a 6 mm cloning cylinder in FAP media at a concentration of 175,000 cells/mm^2^. Cells were fed fresh FAP media every 2–3 days and then fixed with 4% paraformaldehyde after 7 days.

### YAP inhibition assay

FAPs were cultured in FAP media with 0.5 µM Verteporfin (Sigma SML0534) solubilized in DMSO or in 0.1% DMSO alone. FAPs were cultured on collagen-coated polyacrylamide gels and collagen gels at a concentration of 175,000 cells/mm^2^. Cells were fed fresh FAP media every 2–3 days supplemented with Verteporfin or DMSO and fixed with 4% paraformaldehyde after 7 days.

### Immunostaining

Cells were fixed with 4% paraformaldehyde for 15 min, washed in 0.1% bovine serum albumin (BSA) (Fisher BP1600 100) in PBS, and blocked with 5% BSA for 30 min. Samples were immunostained with YAP1 in association with the EdU assay, αSMA in association with the myofibroblast activation assay, or αSMA and Vcam1 on substrates with altered stiffnesses. Samples were stained with a primary antibody overnight in 5% BSA followed by a secondary fluorophore and Acti-stain 555 Phalloidin for 90 min, and Hoechst 33,342 in 0.1% BSA for 15 min. Table 1 indicates the primary antibodies used for immunofluorescence.

**Table 1:** Antibodies used for immunostaining.

Antigen	Vendor	Cat #	Dilution	Host species
αSMA	Fisher	MS113P0	1:800	Mouse
Perilipin	Cell signaling	9349S	1:200	Rabbit
YAP1	Santa Cruz biotechnology	sc-101199	1:1000	Mouse
Vcam1	Abcam	Ab134047	1:500	Rabbit
EdU	Fisher	A10044	1:1000	
Acti-stain	Fisher	50,646,254	1:250	
Hoechst	Fisher	H3570	1:2000	

### Imaging and analysis

Samples of immunostained cells were imaged using an inverted Leica DMi8. Images were captured with a dry 20X/0.40 objective using a Leica DFC9000 GTC camera and LAS X software. At least 200 nuclei were imaged per sample. Each set of samples were captured using the same light intensity and exposure time to ensure consistency during image processing and analysis. Images were analyzed using custom macros in FIJI: ImageJ 2.1.051. To determine proliferation rate, the fraction of EdU+ nuclei was determined. The number of individual nuclei was identified, and an intensity threshold was set for the EdU signal to determine the number of EdU+ nuclei. To perform the myofibroblast activation analysis, the area ratio of αSMA to Acti-stain/F-actin was determined as the signals colocalize on myofibroblasts. To determine YAP localization, individual nuclei were identified, and the YAP intensity of each nucleus was measured as the mean gray value. Each nuclear YAP intensity was divided by the average cytoplasmic YAP intensity to give a nuclear to cytoplasmic ratio. Cytoplasmic area was defined as actin positive and DNA negative area.

Collagen gels were visualized using SHG imaging on a Leica TCS SP8 with a multiphoton laser tuned to 866 nm, using a 25X water immersion objective. Three independent image stacks per gel were taken with a depth of 100 µm and a slice thickness of 1 µm. SHG images were analyzed in FIJI: ImageJ to get the average collagen intensity under αSMA+ or αSMA− cells.

### Statistical analysis

All statistical analysis was done using GraphPad Prism 9.3.1. One-way ANOVA with multiple comparisons or unpaired *t*-tests were used where appropriate to analyze differences between collagen gels. Data was normalized to the control, 3.0 mg/ml unaligned telocollagen gel polymerized at 37 °C. For the polyacrylamide gels, one-way ANOVA with repeated measures using Dunnett’s correction and multiple comparisons were used. Correlation graphs were fitted using a nonlinear regression least squares fit and correlation was done using Pearson Correlation. For SHG image analysis, a two-way ANOVA with multiple comparisons using a Tukey correction was utilized. Significance was set at *p* < 0.05. Data was presented as individual values with mean and standard deviation. Outliers were determined using the Grubb’s test, no data points were excluded.

### Ethics approval and consent to participate

All animal experiments were approved by the University of California Davis Institutional Animal Care and Use Committee.

## Supplementary Information

Supplementary Information.

## Data Availability

The datasets generated during and analyzed during the current study are available from the corresponding author upon reasonable request.

## Abbreviations

αSMA: Alpha smooth muscle actin
ECM: Extracellular matrix
FAP: Fibro-adipogenic progenitor
MSC: Mesenchymal stem cell
MuSC: Muscle stem cell
SHG: Second harmonic generation
YAP: Yes-associated protein
