# Supplementary material for: Matrix stiffness and architecture drive fibro-adipogenic progenitors’ activation into myofibroblasts
Source: Sci Rep. 2022 Aug 9;12:13582. doi: 10.1038/s41598-022-17852-2 (PMC9363488; doi:10.1038/s41598-022-17852-2)
Supplement: Supplementary file 1 — Supplementary Information. [file 41598_2022_17852_MOESM1_ESM.pdf]

Supplemental Figure 1: Softwell Correlation

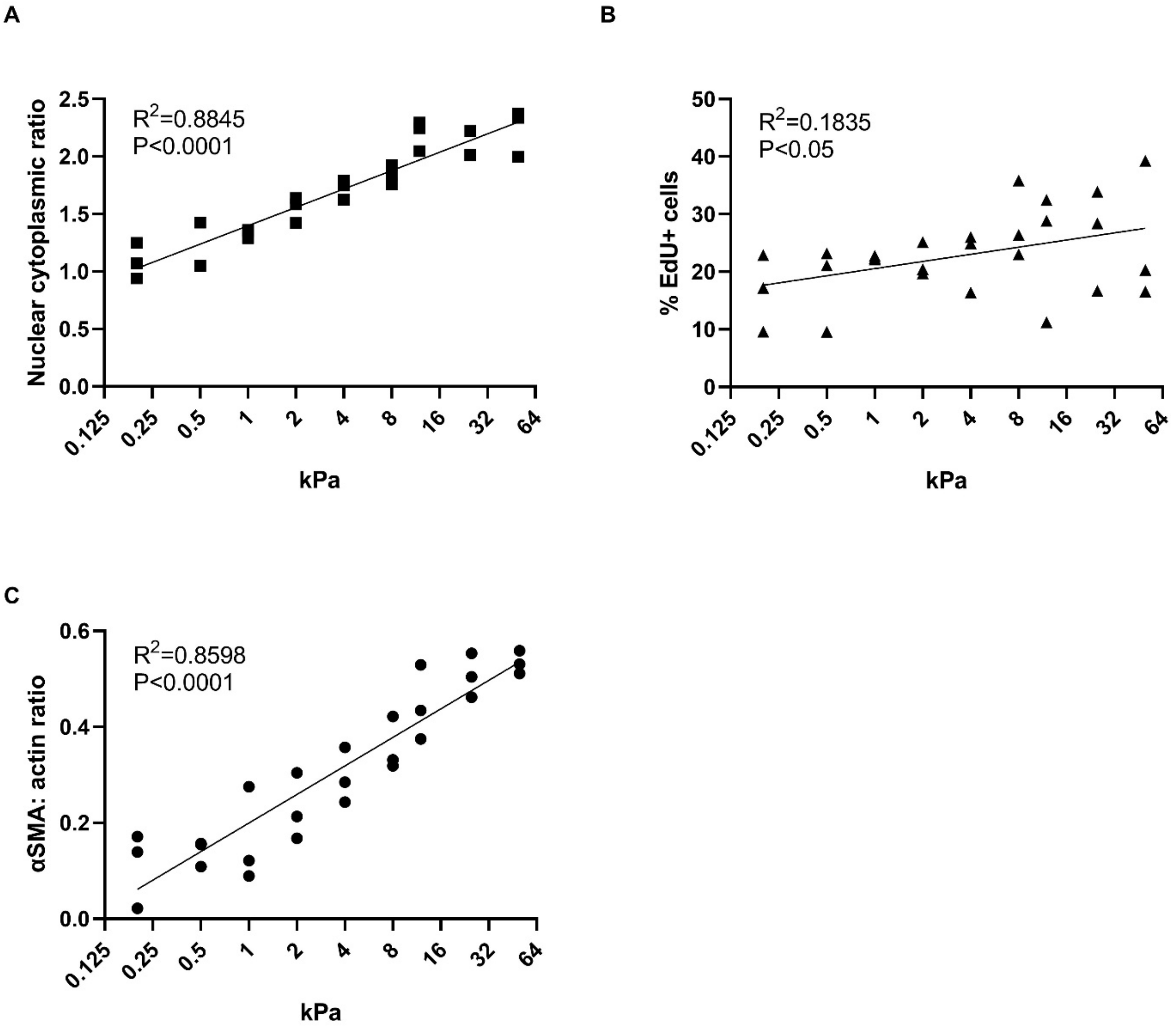

Supplemental Figure 1. Pearson correlation of substrate stiffness with FAP behavior. **A-C)** Correlation of substrate stiffness with YAP nuclear localization, proliferation, and αSMA expression, respectively. Lines fitted with semilog line (x is log2, y is linear) using least squares fit. N=3 mice and n=3 independent gels.

Supplemental Figure 2: Vcam1 Expression and Adipogenesis in FAPs

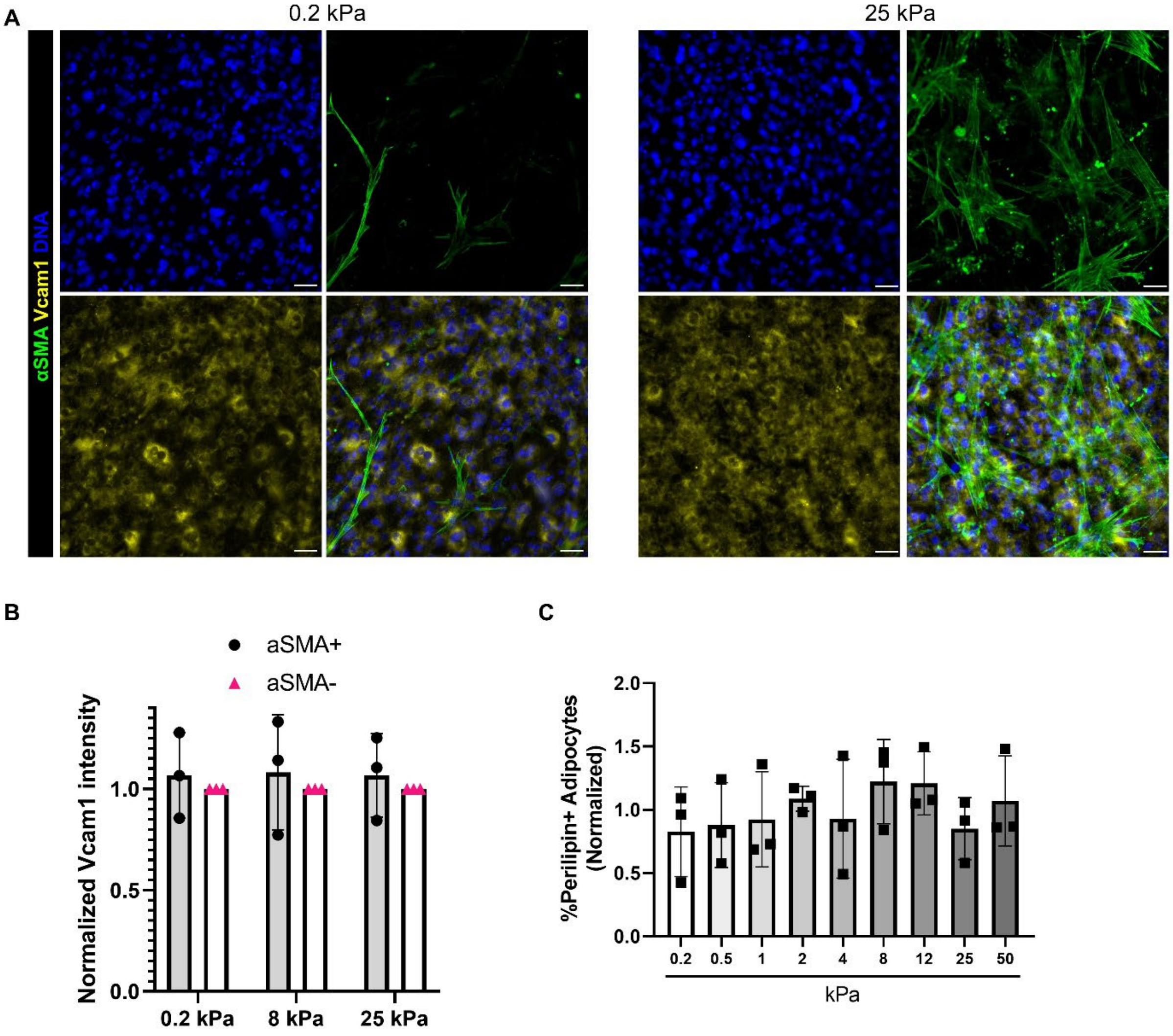

Supplemental Figure 2. Vcam1 Expression in FAPs. **A)** αSMA and Vcam1 expression in FAPs cultured on 0.2 and 25 kPa collagen-coated polyacrylamide gels. **B)** Vcam1 intensity in αSMA+ and αSMA- FAPs, normalized to αSMA- FAP intensity. Scales bars set to 50μm. N=1 mouse and n=3 independent gels. **C)** Number of adipocytes measured by perilipin staining across range of stiffnesses. Data normalized to mean.

Supplemental Figure 3: Nuclear and Cellular Area

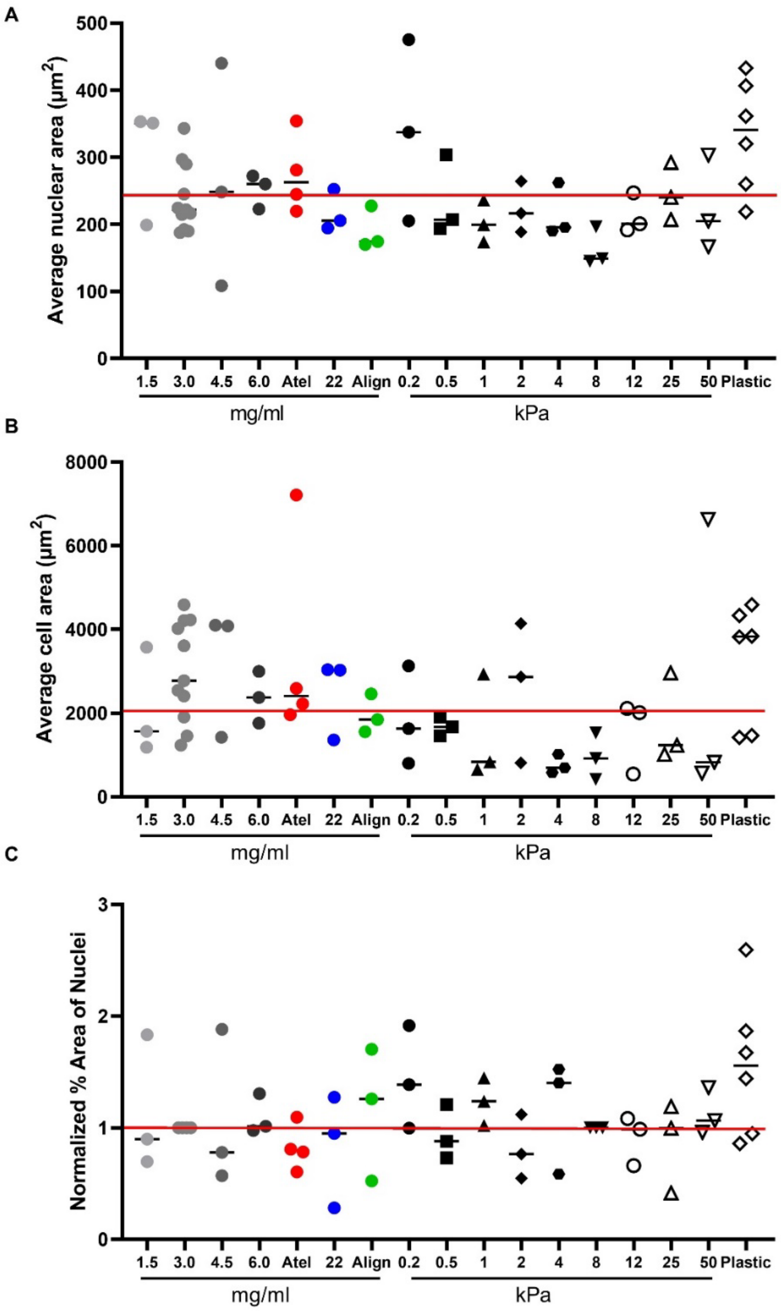

Supplemental Figure 3. Nuclear and Cellular Area. **A)** Average nuclear area across substrate conditions. **B)** Average cellular area across substrates. **C)** Percent nuclear area. Data normalized to experimental control (3mg/ml telocollagen unaligned polymerized at 37°C for collagen gels, 8kPa for polyacrylamide gels). Red line indicates average on graphs. Each data point represents average nuclear area per individual gel. 0.2-50kPa=collagen coated polyacrylamide gels, all other substrates are 3mg/ml telocollagen gel, unless otherwise indicated. Atel=atelocollagen, 22=polymerized at 22°C, Align=aligned with magnetic beads. No significant difference ($p<0.05$) was determined between any of the conditions using a one-way ANOVA with Dunnett's correction.

Supplemental Figure 4: Collagen Alignment

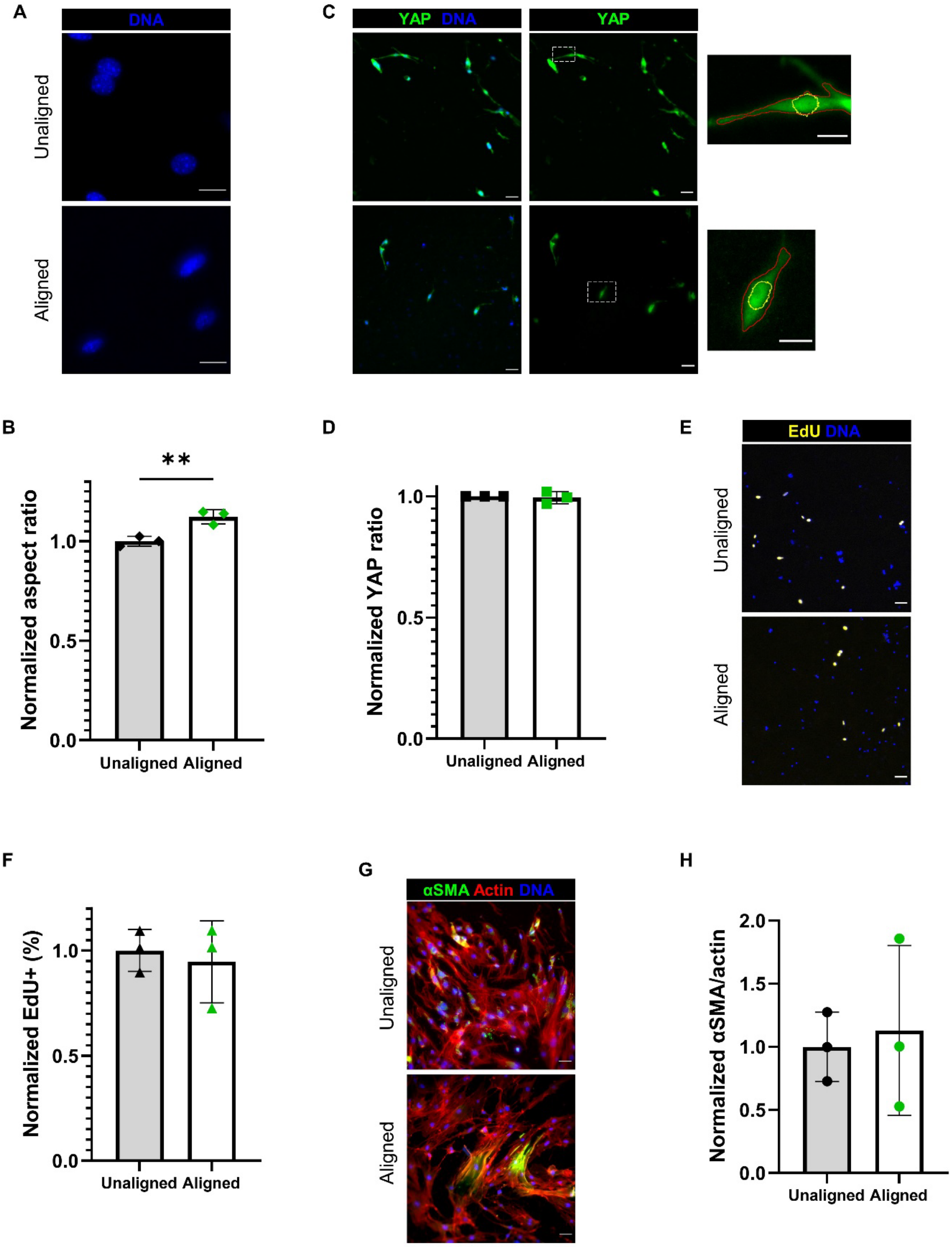

Supplemental Figure 4. FAPs' response to changes in collagen alignment. **A)** Alignment of nuclei with 3mg/ml collagen gels polymerized with or without magnetic bead alignment. N=2 mice and n=3 independent gels. **B)** Quantification of nuclear aspect ratio. **C)** YAP immunofluorescence of FAPs. Yellow outlines indicate nuclei and red outlines indicate cytoplasm on insets. **D)** Quantification of YAP signal intensity inside nucleus and cytoplasm. N=3 mice and n=3 independent gels. **E-F)** EdU signal and quantification of percent proliferating cells after 24 hrs EdU treatment. N=1 mouse and n=3 independent gels. **G)** αSMA signaling in FAPs. **H)** Quantification of αSMA and actin area ratio. N=1 mouse and n=3 independent gels. **$P< 0.01$. Data normalized to unaligned gels. Scales bars set to 50µm, inset scale bars set to 20µm.
